# Nanotechnology Based Approaches for Enhancing Oral Bioavailability of Poorly Water Soluble Antihypertensive Drugs

**DOI:** 10.1155/2016/8525679

**Published:** 2016-04-30

**Authors:** Mayank Sharma, Rajesh Sharma, Dinesh Kumar Jain

**Affiliations:** ^1^School of Pharmacy, Devi Ahilya Vishwavidyalaya, Indore, India; ^2^College of Pharmacy, IPS Academy, Indore, India

## Abstract

Oral administration is the most convenient route among various routes of drug delivery as it offers high patient compliance. However, the poor aqueous solubility and poor enzymatic/metabolic stability of drugs are major limitations in successful oral drug delivery. There are several approaches to improve problems related to hydrophobic drugs. Among various approaches, nanotechnology based drug delivery system has potential to overcome the challenges associated with the oral route of administration. Novel drug delivery systems are available in many areas of medicine. The application of these systems in the treatment of hypertension continues to broaden. The present review focuses on various nanocarriers available in oral drug administration for improving solubility profile, dissolution, and consequently bioavailability of hydrophobic antihypertensive drugs.

## 1. Introduction

Oral drug delivery is the most common, convenient, and extensively used route of administration as it offers advantages like painless administration, no assistance, and patient compliance as compared to other routes such as intramuscular, intravenous, and pulmonary. However, several compounds are unsuccessful and fail in research and development owing to their low absorption and low bioavailability upon oral administration [1]. The drugs with poor oral bioavailability are unable to reach the minimum effective concentration to exhibit therapeutic action [2–4]. Some of the reasons for poor bioavailability are as follows: (a) one of the reasons is poor solubility of drugs that affects the bioavailability as drug should be present in solution form at absorption site; (b) another is inappropriate partition coefficient as it influences the permeation of drug through lipid membrane; (c) first-pass metabolism causes metabolism of drug which results in poor absorption and low bioavailability of the drugs; (d) P-glycoprotein (P-gp) mediated efflux also was shown to alter the pharmacokinetics of drug; the presence of P-glycoprotein in the liver, kidney, and intestine causes reduction in absorption of drug from the gastrointestinal tract and increase in drug elimination; an antihypertensive drug, talinolol, is a P-gp substrate whose oral bioavailability is limited by P-glycoprotein mediated efflux; and (e) degradation of drug in the gastrointestinal tract due to pH of the stomach or enzymatic degradation or by chemical reactions also alters oral bioavailability of drugs [5]. Several approaches have been applied to enhance oral bioavailability of poorly water soluble drugs such as hydrotrophy, solid dispersions, and micronisation. In recent years, nanocarriers are gaining tremendous interest and have shown remarkable advantages over conventional dosage forms in oral drug delivery of hydrophobic drugs [6, 7].

Currently, novel drug delivery systems have increasingly been explored to improve therapeutic efficacy and sustained drug release properties while overcoming the problems like poor solubility and low oral bioavailability of antihypertensive drugs. The available antihypertensive drugs are classified into the following categories: ACE inhibitors, calcium channel blockers, angiotensin antagonist, central sympathomimetics, diuretics, *α*-adrenergic blockers, *β*-adrenergic blockers, and vasodilators. Most of these drugs possess some significant drawbacks like low bioavailability, relatively short half-life, low permeability, and adverse side effects. For effective delivery of these antihypertensive drugs, such drug delivery systems are needed which can provide the following characteristics: (1) low dosing frequency, (2) enhanced bioavailability, (3) increased selectivity, and (4) reduced side effects [8, 9]. Nanotechnology based oral drug delivery systems provide an alternative strategy to administer antihypertensive agents with improved bioavailability and therapeutic effect [10].

## 2. Challenges in Oral Drug Delivery

**Figure 1:**
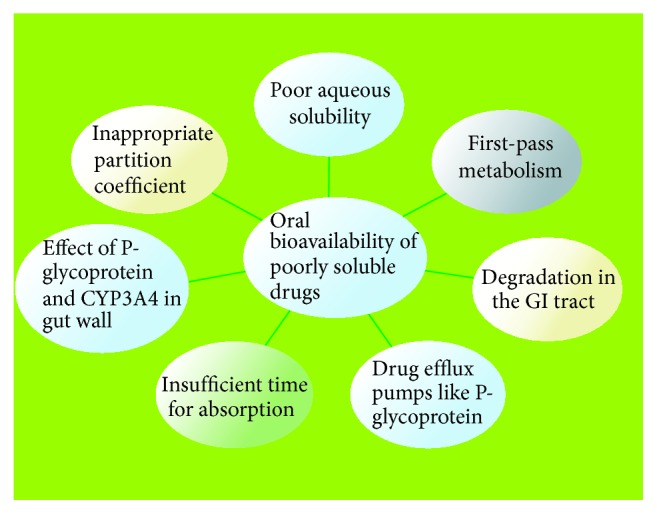
Reasons for poor oral bioavailability of poorly water soluble drugs.

Although the oral drug delivery is effective for drugs with high aqueous solubility and epithelial permeability, efficient oral administration of poorly water soluble drug is a challenge. Presently, most of the new chemical entities are lipophilic and consequently have poor aqueous solubility [11] (Figure 1). On the basis of biopharmaceutical classification system (BCS), a number of new therapeutic entities are characterized under BCS class II (low solubility and high permeability) or BCS class IV (low solubility and low permeability).

Besides, the oral bioavailability of certain drugs is also affected by their poor gastrointestinal permeability. To achieve effective therapeutic action, these drugs have to be given at a high dose like antiviral drugs. Moreover, chemical and enzymatic barriers presented by the gastrointestinal tract (GIT) also affect oral administration of drugs. The change in GIT pH and presence of variety of enzymes significantly affects the oral bioavailability of drugs like antihypertensive, antibiotics, antihyperlipidemic agents, and so forth. Furthermore, drugs with high first-pass metabolism such as repaglinide, *β*-blockers, calcium channel blockers, and ACE inhibitors also have low oral bioavailability. These drugs also present challenge in formulation development for oral administration [12].

## 3. Nanotechnology in Delivery of Poorly Soluble Drugs

Drugs with poor solubility possess difficulty in formulation by applying conventional approaches as they present problems such as slow onset of action, poor oral bioavailability, lack of dose proportionality, failure to achieve steady state plasma concentration, and undesirable side effects. The conventional dosage forms thus may result in over- or undermedication and poor patient compliance [13]. These challenges can be overcome by applying novel drug delivery systems that offer benefits like reduction in dose frequency, lowering of dose size, site specific targeting, enhanced permeability, and improvement in oral bioavailability [14–18]. Nanotechnology is a promising strategy in the development of drug delivery systems especially for those potent drugs whose clinical development failed due to their poor solubility, low permeability, inadequate bioavailability, and other poor biopharmaceutical properties [19–22]. The most common nanotechnology based strategies used in development of delivery systems are nanoemulsions, dendrimers, micelles, liposomes, solid lipid nanoparticles, polymeric nanoparticles, carbon nanotubes, and so forth, which provide controlled, sustained, and targeted drug delivery. The nanotechnology based systems have extensively been investigated for improvement of the bioavailability of antihypertensive drugs [23, 24]. The present review provides an insight of various nanotechnology based approaches having potential in improving oral bioavailability of poorly soluble antihypertensive drugs (Table 1).

## 4. Solid Lipid Nanoparticles (SLNs)

Lipid nanocarriers have gained significant attention in oral drug delivery in the last few decades. The SLNs are submicron colloidal carrier which is composed of physiological lipid, dispersed in water or in an aqueous surfactant solution [46]. SLNs offer benefits such as biocompatibility, nontoxicity, and stability against coalescence. Solid lipid nanoparticles can be applied for delivery of hydrophilic as well as hydrophobic drugs [47, 48]. Venishetty et al. (2012) have shown solid lipid nanoparticles (SLNs) containing carvedilol as a promising strategy to enhance the bioavailability of such poorly soluble drugs. They prepared carboxymethyl chitosan (MCC) coated carvedilol loaded SLNs to improve its bioavailability and to protect it from acidic environment [49]. In another study, Kumar et al. (2007) have demonstrated three to four times increase in bioavailability of nitrendipine solid lipid nanoparticles upon intraduodenal administration. Nitrendipine, an antihypertensive drug, has oral bioavailability between 10 and 20% because of high first-pass metabolism. The solid lipid nanoparticles of nitrendipine were characterized for particle size, zeta potential, drug encapsulation efficiency, and crystalline behavior of lipid and drug. The results of* in vitro* and* in vivo* drug release study indicated solid lipid nanoparticles as a potential carrier for enhancing the bioavailability of nitrendipine [50]. Chalikwar et al. (2014) have shown increase in oral bioavailability of nimodipine, a calcium channel blocker, by formulating solid lipid nanoparticles (SLNs). Nimodipine is highly lipophilic with only 13% bioavailability. SLNs of nimodipine were prepared by 2^3^ factorial design and factors like lipid, surfactant, and cosurfactant concentration were studied. In another investigation, Ekambaram and Sathali (2011) have developed solid lipid nanoparticles (SLNs) of ramipril to increase its bioavailability. Ramipril is a water insoluble drug and its oral bioavailability is only 28%. The SLNs were prepared by employing glyceryl monostearate and glyceryl monooleate with Tween 80, poloxamer*™* 188, and Span 20 as stabilizers. The formulation containing glyceryl monooleate and Span 20 has shown increase in bioavailability [51]. The investigation clearly reveals that SLNs present an effective alternative carrier system for drug delivery. Parmar et al. (2011) have studied* in vitro* and* ex vivo *characteristics of valsartan loaded solid lipid nanoparticles. This study showed SLNs as a promising system to bypass first-pass metabolism, enhance lymphatic absorption, and improve solubility and bioavailability [52].

## 5. Dendrimers

Dendrimers are innovative polymeric carrier attracting attention due to their advantages that include three-dimensional structure, nanometer size, narrow polydispersity index, and controlled molecular structure and are also accompanied with multiple functional groups/multivalency. The word “dendrimers” is derived from a Greek word “Dendra,” which means reminiscent of a tree [53]. Dendrimers have size ranging between 1 and 100 nm with three distinct domains: (i) a core, which is at the center containing atom or a molecule with at least two identical chemical functions; (ii) branches, which are the units repeated in geometric progression that leads to radially concentric layers known as “generations”; and (iii) terminal functional groups, at the surface which determines the properties of dendrimers. Different types of dendrimers are available on the basis of different polymers such as polyamidoamines (PAMAMs), polyamines, polyamides (polypeptides), poly(aryl ethers), polyesters, carbohydrates, and DNA. PAMAM dendrimers are most commonly used. Dendrimers have been applied as a versatile drug delivery system for delivery of drugs, gene, proteins, peptides, and so forth [25, 54]. For different pharmaceutical purposes, dendrimers have also been formed as conjugates by linking to different carriers such as liposomes, CNTs, and nanoparticles [55]. Some of the applications of dendrimers include solubilization, gene therapy, and immunoassay. However, the conjugation and encapsulation of drug with dendrimers have provided a platform for oral delivery of hydrophobic drugs like antihypertensive drugs or anticancer drugs [29, 42, 56, 57].

The improvement in solubility through dendrimers depends upon dendrimer concentration, pH, generation size, core, terminal functionality, and temperature. Candesartan cilexetil is a calcium channel blocker used in the treatment of hypertension. The permeability of candesartan cilexetil depends on its aqueous solubility and lipid-protein partition coefficient in relation to the stratum corneum. In an investigation, Gautam and Verma (2012) fabricated polyamidoamine (PAMAM) dendrimers containing candesartan cilexetil. The results of investigation have showed significant increase in water solubility of candesartan cilexetil in the form of PAMAM dendrimers [58]. Dendrimers of lomerizine, a calcium channel blocker, have also been developed to increase its aqueous solubility [59].

The studies also suggest that conjugation of drug to dendrimer enables bypassing of the efflux transporter which leads to increase in drug solubility and therefore increasing drug bioavailability. In a study, propranolol (a calcium channel blocker), a known substrate of the P-glycoprotein (P-gp) efflux transporter, has been conjugated to lauroyl-G3 dendrimers. This conjugated propranolol has shown increased solubility [60]. PAMAM dendrimers have significantly enhanced the water solubility of nifedipine [61]. However, the application of dendrimers in oral delivery is in its infancy but may emerge as a rewarding strategy in the future [54, 62].

## 6. Nanosuspensions

**Figure 2:**
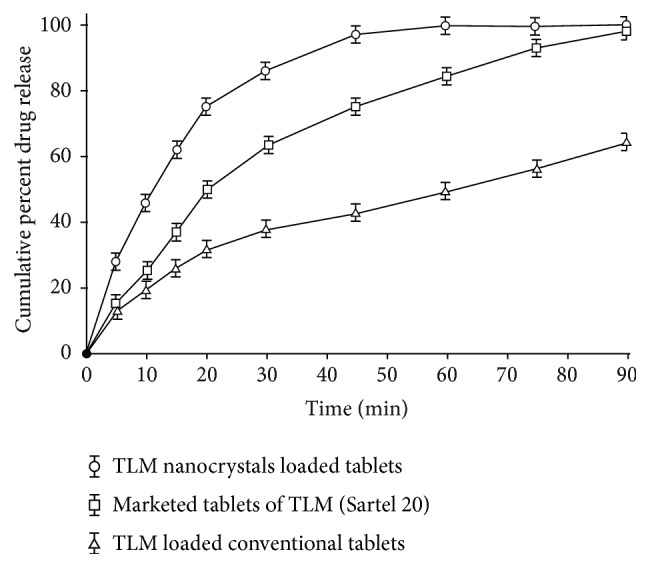
Comparison of* in vitro* drug release profiles of Telmisartan (TLM) loaded nanosuspension, marketed formulation, and conventional tablets.

Nanosuspensions are biphasic, colloidal dispersions of drug particles which are stabilized by using surfactants. Nanosuspensions contain particles dispersed in an aqueous vehicle with the size of particle less than 1 *μ*m. Nanosuspensions can overcome the problems related to the delivery of poorly water soluble drugs due to their nanosize particle range. Nanosuspensions can be formulated with high solid content up to 40%, which reduces their dose size and improves patient compliance. Various methods such as spray drying, freeze drying, and extrusion-spheronization have been applied for converting nanosuspensions to pellets/tablet-like dosage forms. Patel et al. (2014) have described an approach to develop a nanosuspension of a poorly water soluble antihypertensive drug to improve the bioavailability. Further, the optimized batch of nanosuspension was converted into a solid dosage form. Telmisartan (TLM) was selected as a model drug. The TLM loaded nanosuspension was optimized by applying 3^2^ full factorial design. The concentration of stabilizer and amount of milling agents were taken as principal component of analysis (PCA). Lyophilization process was used to develop tablets of nanosuspension. The* in vitro* drug release study was carried out over optimized batch of TLM loaded tablets, marketed tablets (Sartel® 20), and conventional tablets in 0.1 M HCl as a dissolution medium. The results of* in vitro *drug release have demonstrated higher value of cumulative percentage release (CPR) for nanosuspension loaded tablet formulation in comparison to two other formulations as shown in Figure 2 [63]. The* in vivo* pharmacokinetic study was performed in TLM loaded tablets against marketed tablets in Wistar rats and also revealed improvement in rate of absorption from nanosuspension. Thus, the study indicated improvement in rate and extent of oral absorption of TLM from nanosuspension loaded tablets as compared to marketed formulations. This effectiveness was attributed to nanometer size of particles in nanosuspension with subsequent increase in surface area and absorption.

The other properties of nanosuspensions like rapid onset of action, enhanced surface area, higher level of saturation solubility, and higher adhesiveness to gastrointestinal epithelium also lead to improved oral absorption and bioavailability of lipophilic drugs. Liu et al. (2014) have shown increased oral bioavailability of carvedilol by developing its osmotic pump capsule. The nanosuspension of carvedilol was first prepared and transformed into osmotic pump capsule using semipermeable capsule shells. The* in vivo* studies on beagle dogs have shown significant improvement in the bioavailability as compared to marketed products [64]. The nanosuspensions also are reported to have an excellent disintegration profile which increases dissolution rate with complete dissolution in minutes [39]. Thadkala et al. (2015) have demonstrated improvement in dissolution rate and consequently absorption of nebivolol hydrochloride by developing its oral nanosuspension tablets. Nebivolol hydrochloride is a lipophilic drug which is *β*1 receptor antagonist and comes under class II in BCS. The nanosuspension of nebivolol was prepared by solvent displacement/nanoprecipitation method. The* in vitro* dissolution studies of optimized formulation showed maximum drug release of 98.93% within 15 minutes which followed first-order release kinetics. The* in vitro* drug release of optimized formulation was compared with the* in vitro* drug release of innovator product (Nebilet) that showed drug release of 98.37% within 60 minutes and of pure drug 27.34% within 60 minutes. The results of the study concluded that nanosuspension formulation has shown increased drug release rate as compared to innovator product (Nebilet) and pure drug [65].

One important challenge in fabricating nanosuspensions for oral administration is particle size of the nanosuspension and stability during storage. However, proper selection of the surfactants and/or stabilizers and the method of preparation can produce physically stable nanosuspensions with long-term storability. Rajalakshmi et al. (2012) have shown nanosuspensions as a promising alternative strategy with improved stability and biopharmaceutical efficacy for poorly water soluble drugs. They prepared valsartan nanosuspension by employing soya lecithin and poloxamer as stabilizers. The* in vitro* drug release study showed higher release from nanosuspension [66]. In a pioneer study, Sahu and Das (2014) have reported the increased solubility and oral bioavailability of felodipine in the form of physically stable nanosuspension [36]. A report of another study on the candesartan cilexetil has also revealed nanosuspension system as an effective alternative strategy to improve oral bioavailability of poorly water soluble antihypertensive drugs. The results of this study have shown significant lowering in high blood pressure upon administration of nanosuspension as compared with plain drug suspension and, thus, the investigation demonstrated significant increase in antihypertensive activity of candesartan in the form of nanosuspension. The study also showed that nanosuspensions systems could be effectively transformed from laboratory scale to pharmaceutical market and could be an effective approach to increase bioavailability for poorly soluble drugs, especially when drugs are simultaneously insoluble in organic as well as aqueous media [67].

## 7. Nanoemulsions

Nanoemulsions are oil-in-water (o/w) emulsions with droplet size in the range of 100 and 500 nm. Nanoemulsions provide advantages of solubilization of hydrophobic molecules in the oily phase, modification of oil droplets with polymers to prolong circulation time, and targeting tumors passively and/or targeting ligands actively. Most commonly used methods to prepare nanoemulsions are low-energy emulsification and high-energy emulsification [68]. Ghai and Sinha (2012) have developed nanoemulsions as emulsified drug delivering carrier for selective *β*-1 adrenoreceptor blocker of talinolol. The talinolol nanoemulsion is comprised of 5% (w/v) Brij-721 ethanolic solution, triacetin, and water in ratio of 40 : 20 : 40 (%w/w). The droplet size, polydispersity index, surface morphology, and* in vitro* and* in vivo* release of nanoemulsion were investigated. The results of the study have revealed significant increase in drug release and bioavailability, which showed increase in solubility of drug from nanosized emulsion [69]. Gorain et al. (2014) have proved the nanoemulsion as a promising approach for increasing bioavailability. Olmesartan medoxomil (OM) was selected as a model drug. The oil-in-water (o/w) nanoemulsion of OM was prepared using soya bean oil, sefsol 218, and Solutol HS15. The physicochemical characterization has shown that the nanoemulsion was thermodynamically stable with nanometer droplet size, low polydispersity index, and increase in permeability from the CaCo-2 cell line. The pharmacokinetic study results showed 2.8-fold increase in area under curve (AUC_0–27_) which has enhanced control over hypertension and has led to 3-fold reduction in dose [26]. Guan et al. (2014) have compared the pharmacokinetic properties of nitrendipine submicron emulsion with conventional nitrendipine solution in rats. The ultra performance liquid chromatography coupled with mass spectrometry detection (UPLC-MS/MS) was applied for analysis of plasma concentration. The AUC, *C*_max_, and *t*_1/2_ for nitrendipine emulsion and nitrendipine solution were found to be 900.76 ± 186.59 versus 687.08 ± 66.24 ngh/mL, 854.54 ± 159.48 versus 610.59 ± 235.99 ngh/mL, and 2.37 ± 1.99 versus 2.80 ± 2.69 h. In comparison to nitrendipine solution, the nanoemulsion showed improved bioavailability and therapeutic efficacy [70].

## 8. Self-Nanoemulsifying Drug Delivery System (SNEDDS)

**Figure 3:**
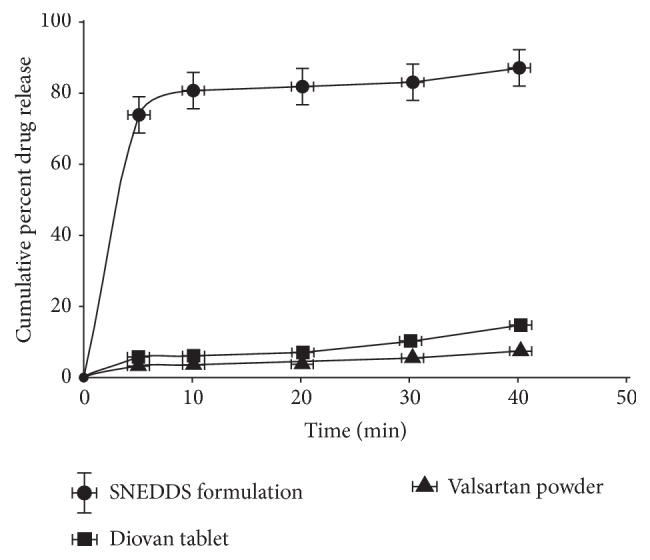
Comparison of* in vitro* drug release profile of SNEDDS containing valsartan powder with valsartan tablet.

SNEDDS are nanoscale oil-in-water (O/W) nanoemulsion, available in the form of anhydrous isotropic mixture of surfactant, oil, and drug, which when introduced into aqueous phase with gentle agitation get converted into nanoemulsion. The digestive motility of gastrointestinal tract provides required agitation for formation of nanoscale emulsions. The SNEDDS retain benefits associated with nanoemulsions like increased oral bioavailability, increased permeation of drug, improved chemical and enzymatic stability, and ease of fabrication and scale-up. The SNEDDS of poorly water soluble drug have shown improvement in solubility. Rajinikanth et al. (2012) have shown significant increase in dissolution rate of valsartan by forming its SNEDDS. The SNEDDS of valsartan was developed using Labrasol (oil), Tween 20, and PEG 400. The investigation data showed desirable zeta potential, stability, droplet size, and sixfold increase in drug release as compared to marketed valsartan tablet and powder. From the results, it may be concluded that SNEDDS of poorly soluble drugs presents a promising drug formulation system for oral delivery of antihypertensive drugs (Figure 3) [71]. Similarly, another investigation has also shown the improvement in the drug release of valsartan and olmesartan in the form of SNEDDS [30, 34].

## 9. Polymeric Nanoparticles

Polymeric nanoparticles are colloidal drug delivery carriers with particle size of 10 to 100 nm. The main advantages of nanoparticles are (1) enhanced bioavailability, (2) increased specificity and targeting to desired site, and (3) reduced toxicity and dose. All these benefits enable safe delivery of drugs especially to target sites without affecting normal tissue. Polymeric nanoparticles have been successfully developed for different application such as for tumor targeting and gene delivery [40]. These nanoparticles are prepared by employing biodegradable, synthetic, and natural polymers. The therapeutic effect of polymeric nanoparticles significantly depends on drug release and biodegradation of polymers. The drug release from nanoparticles follows diffusion mechanism, erosion mechanism, or both erosion and diffusion in combination [72]. Some of the most commonly employed polymers in the development of nanoparticles are poly(D,L-lactide-co-glycolide), polycaprolactones, and poly(D,L-lactide) [31, 43, 73–76]. The polymer molecular weight, method of preparation, particle size, and type of stabilizer significantly affect the oral drug delivery through nanoparticles. The polymeric nanoparticles can be effectively employed for increasing oral bioavailability of drugs with poor solubility, chemical/enzymatic stability, and poor permeability.

The investigation of valsartan loaded nanoparticles has shown prolonged release of drug and thereby decreases its dose size, frequency of dose, and side effects [77]. Moreover, the therapeutic use of isradipine, an antihypertensive agent (a calcium channel blocker), is hampered due to its rapid and intense vasodilator effect. But isradipine in its nanoparticles form has shown initial slow release and prolonged antihypertensive effect, when given orally [78]. For oral bioavailability of hydrophobic drugs, they must be dissolved in gastrointestinal fluids. Zhang et al. (2010) have synthesized mesocellular foam (MCF) nanoparticles of Telmisartan. The MCF are formed with a continuous 3D pore system using Pluronic 123 as a surfactant coupled with cetyl trimethyl ammonium bromide (CTAB) as a cosurfactant. Telmisartan in mesocellular form has shown high drug loading and high dissolution rate [79]. One of the widely used antihypertensives, nebivolol, presents problem of poor solubility and bioavailability and thus frequent dosing. Jana et al. (2014) have studied nanoparticles of nebivolol by employing Eudragit RS 100. The nanoparticles were developed by solvent evaporation process. Fourier transform infrared spectroscopy and differential scanning calorimetry (DSC) studies have shown drug-polymer compatibility. The* in vitro* drug release data have shown prolonged drug release with low initial burst release [80]. Zhu et al. (2014) have developed felodipine loaded poly(l-lactic acid) nanoparticles. Felodipine is a second generation of 1,4-dihydropyridines (1,4-DHPs) and has much less solubility in water and thus low bioavailability. The nanoparticles were prepared by emulsion solvent evaporation technique. The investigation results have shown controllable drug release and effective* in vitro* compatibility [81]. Kumar et al. (2015) have shown improved oral bioavailability of valsartan by preparing its nanoparticles. The nanoparticles were optimized and developed by full factorial design [82]. A report of* in vitro* and* in vivo* study of nifedipine nanoparticles also showed increased patient compliance by decrease in frequency of administration. The polymeric nanoparticles have shown increase in antihypertensive activity owing to its improved oral bioavailability and long-lasting action [83].

## 10. Carbon Nanotubes (CNTs)

Presently, carbon nanotubes are gaining tremendous attention as novel drug delivery carriers. CNTs offer features like (1) high cellular uptake, (2) enhanced transmembrane penetration accumulation due to needlelike shape of CNTs, and (3) ability of high drug loading owing to their increased surface area. Several* in vivo *and* in vitro* studies on CNTs have proven them to be an effective drug delivery system. The poly(amidoamine)- (PAMAM-) functionalized multiwalled carbon nanotubes (MWNTs) loaded with poorly water soluble carvedilol were developed to improve the drug loading capacity and dissolution. PAMAM-MWNTs have shown marked increase in solubility. From the study, it is concluded that MWNTs present a larger surface area for proper dispersion of drug and PAMAM modifies drug loading capacity and improves the solubility of drugs [84]. However, despite these advantages in drug delivery, CNTs are still not considered safe for clinical application [85].

## 11. Polymeric Micelles

Micelles are a group of amphiphilic surfactant molecules that rapidly aggregate into a spherical vesicle in water and range from 10 to 100 nm in size. Micelles arrange themselves in a spherical form upon contact with aqueous solutions. The center of the micelle is hydrophobic and thus entrapment of lipophilic drug is easily possible. Polymeric micelles are formed of block copolymers consisting of hydrophilic and hydrophobic monomer units. Several polymers in combination are used in formation of micelles, for example, poly(ethylene oxide) (PEO) and poly(propylene oxide) (PPO), poly(lactic acid) (PLA), or other polyethers or polyesters. PEO–PPO–PEO structure with the trade names of Pluronic® and poloxamer is one of the commonly used polymers in micelles formulation. Polymer micelles have benefits like enhanced drug solubility, sustained circulation half-life, specificity at target sites, and reduced toxicity [28, 32, 86, 87]. Satturwar et al. (2007) have reported increase in drug loading capacity and drug release by fabricating PEG-b-poly(alkyl(meth)acrylate-co-methacrylic acid) micelles containing poorly water soluble candesartan cilexetil [35].

Polymeric micelles can also be used for targeted drug delivery, which is mediated by increased permeability and retention behavior, by forming micelles of stimuli-responsive amphiphilic block copolymers or by conjugating specific targeting ligand molecules to the surface of micelles [33]. In a study, poly(epsilon-caprolactone)-poly(ethylene oxide)-poly(epsilon-caprolactone) amphiphilic triblock copolymer micelles were studied for drug release behavior. Nimodipine was selected as a hydrophobic model drug. The result suggested that, with the increase in ratio of poly(epsilon-caprolactone), the size for micelle and drug loading capacity increased whereas the drug release rate decreased [37]. The polymeric micelles have shown the ability to improve oral delivery and to enhance the therapeutic efficacy of poorly water soluble drugs [27, 28].

## 12. Nanocrystals

Nanocrystals are comprised of aggregates of large number of atoms with size between 10 nm and 400 nm. The steps involved in formation of nanocrystals involve formation of nanosuspension, followed by wet milling, high pressure homogenisation, nanocrystallisation, and finally spray drying to obtain nanosized crystals [88]. The decrease in drug particle size to nanoscopic crystals results in an increased surface area to volume ratio [89]. Hecq et al. (2005) have investigated the nanocrystals of nifedipine for enhancement of solubility and dissolution rate. This study indicated that there was retention of crystalline state upon particle size reduction and improvement in dissolution rate of nifedipine [90]. Surface modification of nanocrystals also plays an important role in* in vitro* and* in vivo* behavior of nanocrystals. In a study, chitosan (positively charged polymer) was employed to modify surface of nanocrystals containing nitrendipine (negatively charged drug). The investigation results of modified nanocrystals showed improvement in physical stability and demonstrated remarkable improvement in bioavailability as compared to traditional dosage form. The physical stability of the chitosan modified nanocrystals was remarkably improved under ambient conditions. On the basis of experimental data, it can be concluded that surface modification of the nanocrystals with some polymer would emerge as an efficient method for controlling* in vitro* and* in vivo* performance of the nanocrystals and therefore increasing the bioavailability of poorly water soluble drugs [91]. An investigation of nitrendipine nanocrystals has also revealed increase and* in vitro* drug release profile. The* in vivo* study has shown 15-fold and 10-fold increase as compared to physical mixture and commercial tablet, respectively [41]. A study of nimodipine nanocrystals has also suggested nanocrystals as a successful delivery system for hydrophobic drugs [92].

## 13. Proliposomes

Proliposomes are defined as dry, free-flowing particles with a dispersed system that can immediately form a liposomal suspension when in contact with water. Compared with conventional liposomes, proliposomes exhibit more advantages in promoting drug absorption. Because of their solid properties, the physical stability of liposomes can be improved without influencing their intrinsic characteristics. Therefore, proliposomes would be a potential vehicle to help improve the oral absorption of hydrophobic drugs [93]. Isradipine-loaded proliposomes were developed to enhance the oral bioavailability and were compared with its oral suspension. The pharmacokinetic parameters of isradipine proliposomes were evaluated in male albino Wistar rats and 2.4 times increase in bioavailability was found from the optimized proliposome batch as compared to control oral suspension [94]. Kim et al. (2008) have shown potential of proliposomal formulation in sustained drug delivery of propranolol. The characterization of proliposomal formulation has shown good flowability, particle size distribution, and well conversion into liposomes by hydration and desirable* in vitro* drug release [95]. Similarly, researchers have reported significant enhancement in the oral bioavailability of valsartan loaded proliposomes [96]. Furthermore, several patents for preliposomal formulations are also available which contain poorly water soluble antihypertensive agents.

## 14. Mesoporous Silica Nanoparticles (MSNs)

Mesoporous silica nanoparticles (MSNs) have emerged as a promising novel drug delivery vehicle due to their attractive properties like pore size, high drug loading, porosity, and controlled release kinetics. MSNs are applied as delivery reagent due to chemical properties, thermal stability, and biocompatibility of silica, which enables controlled drug delivery to target site [97]. Zhang et al. (2012) have investigated the influence of MSNs in improving oral bioavailability of poorly water soluble Telmisartan (TEL), along with its cellular uptake and cytotoxicity. The cellular uptake study was carried out by laser scanning confocal microscopy, transmission electron microscopy, and fluorescence activated cell sorting. A human colon carcinoma (CaCo-2) cell line and beagle dogs were employed to study drug permeability and* in vivo* drug release, respectively, against marketed formulation Micardis. The report of the investigation has indicated high cellular uptake depending on concentration and size along with high drug permeability, dissolution rate, and* in vivo* pharmacokinetics of poorly soluble TEL as compared to marketed formulation. Thus, the study has shown the potential of MSNs in enhancing oral bioavailability of poorly water soluble drug [38]. In order to control the drug release, Alexa et al. (2012) have extensively investigated the effectiveness of MSNs on drug release profiles of two antihypertensive drugs, captopril and aliskiren. In the study, pure silica (SBA-15) and MgO-modified mesoporous silica (MgO/SBA-15) were used as vehicles to evaluate encapsulation and controlled release of drugs. The X-ray diffraction pattern, Fourier transform infrared spectra, scanning electron microscopy, and ultraviolet spectroscopic measurements were performed on formulations. The results have shown that MgO/SBA-15 samples have more affinity with drug molecules than pure silica SBA-15, which could be due to basicity of magnesium oxide. The* in vitro* release profile has revealed gradual drug release for longer period from both SBA-15 and MgO/SBA-15 formulation [44]. There are various types of mesoporous silica and the most commonly investigated as drug carrier are the channel-like MCM-41 and SBA-15 [45]. Hu et al. (2012) have investigated a unique 3D structured SBA-16 as a carrier for poorly water soluble carvedilol against MCM-41 and its corresponding crystalline form for drug loading and drug release behavior. The drug release study has demonstrated enhanced dissolution rate with both SBA-16 and MCM-41 form as compared to its crystalline form. The increase in dissolution rate was attributed to increased surface area of bonded CAR, its noncrystalline state, and hydrophilic nature of silica. Moreover, as compared to MCM-41, SBA-16 has shown faster release indicating its potential as a carrier for delivery of poorly soluble drugs [98].

## 15. Conclusion

Nanotechnology holds a great potential in effective delivery of poorly soluble antihypertensive drugs by improving solubility and oral bioavailability. Moreover, novel drug delivery approaches have appeared as strategies to revitalize the development of new hydrophobic entities. The biocompatibility, colloidal size, drug targeting, lowered dose size, reduced toxicity, and patient compliance are some important advantages of nanosystems. Literature survey reveals various benefits of novel drug delivery system, some of the advantages include improved targeting, bioavailability, therapeutic efficacy, and production scalability provided by solid lipid nanoparticles, whereas SNEDDS provides enhanced interfacial area for drug partitioning and improved bioavailability and does not require high-energy emulsification; thus it reduces the manufacturing cost. Besides, polymeric nanoparticles provide ease of manipulation of particle size and surface characteristics for both active and passive targeting. Dendrimers have gained interest due to their unique properties like highly branched structure, multivalency, and versatile chemical compositions. Proliposomes contain free-flowing granular material which results in enhanced solubility, improved stability, and ease of handling. However, though liposomes provide controlled drug release and increased bioavailability but show tendency to aggregate or fuse. Although significant advancements are made in nanotechnology, some challenges have been encountered in the development of novel drug delivery systems like (a) transformation of these nanocarrier systems from laboratory scale to pharmaceutical market, (b) depending on factors like cost of fabrication, reproducibility of properties of formulation on production scale, and (c) benefits to human population owing to large variation in pharmacokinetics. However, despite these challenges, the development and benefits offered by novel drug delivery systems cannot be ignored. Thus, nanotechnology offers opportunity for formulation scientists to extend research and development to overcome the challenges related with current antihypertensive drugs, thereby improving the patient compliance and therapeutic efficacy.

## Figures and Tables

**Table 1:** Summary of some of the investigations on nanosystems of antihypertensive drugs.

Name of drug	Colloidal system	Application	Ref. number
Carvedilol	Solid lipid nanoparticles	Enhanced bioavailability and protecting it from acidic environment	[25]
	Nanosuspensions	Increased oral bioavailability	[26]
	Carbon nanotubes	Drug loading capacity and improving the solubility	[27]
	Mesoporous silica nanoparticles	Improvement in drug loading and drug release profile	

Nebivolol	Polymeric nanoparticles	Prolonged drug release	[28]

Valsartan	Solid lipid nanoparticles	Bypassing first-pass metabolism, enhancing lymphatic absorption, and improving solubility and bioavailability	[29]
	Nanosuspensions	Enhanced drug release	[30]
	Self-nanoemulsifying drug delivery system	Increase in dissolution rate	[31]
	Polymeric nanoparticles	Prolonged release of drug and thereby it decreases its dose size, frequency of dose, and side effects	[32]
	Proliposomes	Good flowability and particle size distribution and well conversion into liposomes by hydration and desirable *in vitro* drug release	[33]

Felodipine	Nanosuspensions	Enhanced solubility and oral bioavailability	[34]
	Polymeric nanoparticles	Controllable drug release and effective *in vitro* compatibility	[35]

Nifedipine	Dendrimers	Enhanced water solubility	[36]
	Polymeric nanoparticles	Improved oral bioavailability	[37]
	Nanocrystals	Enhanced dissolution rate	[38]

Candesartan cilexetil	Dendrimers	Improved water solubility	[39]
	Nanosuspension	Improved bioavailability	[40]
	Polymeric micelles	Increased drug loading capacity and drug release	[41]

Nitrendipine	Solid lipid nanoparticles	Enhanced bioavailability	[42]
	Nanoemulsion	Improved therapeutic efficacy and bioavailability	[43]
	Nanocrystals	Improvement in physical stability, *in vitro* drug release, and bioavailability	[44, 45]
